# Radiologie in der stadiengerechten Beurteilung kolorektaler Karzinome

**DOI:** 10.1007/s00117-025-01458-6

**Published:** 2025-05-19

**Authors:** Sophia Wirth, Nino Bogveradze, Katharina Lampichler

**Affiliations:** 1 Present Address: Universitätsklinik für Allgemeine Radiologie und Kinderradiologie, Währinger Gürtel 18–20, 1090 Wien, Österreich; 2 American Hospital Tbilisi, Tiflis, Georgien

**Keywords:** Dickdarmkrebs, Primäre Stadieneinteilung, Computertomographie, Magnetresonanztomographie, TNM-Klassifikation, Colorectal cancer, Primary staging, Computed tomography, Magnetic resonance imaging, TNM classification

## Abstract

**Hintergrund:**

Die Bildgebung, insbesondere die Magnetresonanztomographie (MRT), ist seit Langem die Grundlage für die Stadieneinteilung des Rektumkarzinoms. Im Gegensatz dazu ist die Computertomographie (CT) der Standard für die Stadieneinteilung beim Kolonkarzinom. Anhand der primären Stadieneinteilung werden eine individuelle Risikostratifizierung und Therapieplanung durchgeführt.

**Ziel:**

Zusammenfassung der aktuellen internationalen Richtlinien zur primären Stadieneinteilung unter Berücksichtigung der Vor- und Nachteile unterschiedlicher bildgebender Methoden.

**Material und Methode:**

Basierend auf einer umfangreichen Literaturrecherche wird der aktuelle Wissensstand im Bereich der Stadieneinteilung von kolorektalen Karzinomen zusammengefasst.

**Ergebnisse:**

Die TNM-Stadieneinteilung umfasst die lokale Tumorausdehnung und das Vorhandensein von Lymphknoten- und Fernmetastasen. Für die lokale Tumorausdehnung ist die MRT beim Rektumkarzinom sehr gut geeignet. Beim Kolonkarzinom gibt es in der CT deutliche Einschränkungen hinsichtlich der Sensitivität für die Unterscheidung einzelner Stadien. Lymphknotenmetastasen können eine große Herausforderung für beide Bildgebungsmethoden sein, daher wurden mehrere Malignitätskriterien definiert. Die Detektion von Fernmetastasen ist mit Ausnahme von Lebermetastasen weiterhin die Domäne der CT.

**Schlussfolgerung:**

Je nach Tumorlokalisation werden bei der primären Stadieneinteilung von kolorektalen Karzinomen entweder MRT oder CT empfohlen, auch eine Kombination von beiden Modalitäten kann sinnvoll sein. Die genaue Beurteilung des Primärtumors, der Lymphknoten und der Organe mittels Bildgebung ist ein essenzieller Bestandteil im Therapiekonzept von kolorektalen Karzinomen.

## Abstract (en)

**Background:**

Imaging, specifically magnetic resonance imaging (MRI), has long been the cornerstone for staging rectal cancer. In contrast, computed tomography (CT) is the standard for colon cancer staging. Primary staging is essential for individualized risk stratification and therapy planning.

**Objectives:**

To summarize current international guidelines on primary colorectal cancer staging, taking into account the advantages and disadvantages of different imaging methods.

**Materials and methods:**

Based on extensive literature research, the current state of knowledge in the staging of colorectal cancer is summarized.

**Results:**

TNM staging includes local tumor extension as well as the presence of lymph node and distant metastases. For local tumor extension, MRI is very well suited for rectal cancer. In colon cancer, CT shows significant limitations regarding the sensitivity of differentiating individual stages. Lymph node metastases can pose a major challenge for both MRI and CT, which has led to the definition of several malignancy criteria. The detection of distant metastases remains primarily within the domain of CT, except for liver metastases.

**Conclusions:**

Depending on tumor location, either MRI or CT is recommended for primary staging of colorectal cancer, and a combination of both modalities may also be appropriate. Accurate assessment of the primary tumor, lymph nodes, and organs using imaging is an essential component of the therapeutic approach to colorectal cancer.

Weltweit ist das kolorektale Karzinom (KRK) die dritthäufigste Krebsdiagnose, wobei sich aus derzeit noch unerklärlichen Gründen eine steigende Inzidenzrate in einer jüngeren Alterspanne abzeichnet [6, 8, 30]. Abhängig vom Stadium bei Erstdiagnose, gibt es deutliche Unterschiede in der 5‑Jahres-Überlebenswahrscheinlichkeit. Diese beträgt in Stadium I 93,6 %, in Stadium IV jedoch lediglich 13,4 % [8].

Zur primären, radiologischen Stadieneinteilung wird sowohl beim Kolonkarzinom als auch beim Rektumkarzinom auf das TNM-System zurückgegriffen. Dabei steht T für Tumor, N für Node (Lymphknoten) und M für Metastasen. Da das Kolonkarzinom oft ein anderes Management als das Rektumkarzinom mit sich bringt, bestehen gewisse Unterschiede in der Stadieneinteilung und Therapieplanung. Vor allem beim Rektumkarzinom wird je nach individuellem Risikoprofil in der präoperativen Bildgebung ein risikoangepasstes Behandlungskonzept erstellt – von alleiniger Operation bis hin zu neoadjuvanter Chemo- und/oder Strahlentherapie. Entsprechend werden diese beiden Karzinomtypen im folgenden Artikel getrennt erläutert.

## Kolonkarzinom

### CT-Protokoll

Sollte der Verdacht auf ein Kolonkarzinom bestehen oder bereits eine histologische Diagnosesicherung vorliegen, wird zur weiteren Therapieplanung laut deutschen aber auch internationalen Leitlinien eine kontrastmittelunterstütze Computertomographie (CT) durchgeführt [18, 19, 21, 22, 24]. Mittels CT kann sowohl die lokale Ausdehnung des Tumors als auch das Vorhandensein von Metastasen beurteilt werden. Das entsprechende CT-Protokoll variiert von Zentrum zu Zentrum. Die Durchführung einer CT Thorax und Abdomen mit arterieller Phase des Oberbauchs und venöser Phase von Thorax und Abdomen deckt sämtliche Metastasierungswege ab, und die arterielle Phase ermöglicht eine genaue Darstellung der Blutgefäße und erleichtert die Charakterisierung möglicher Leberläsionen. Zur präziseren Beurteilung des lokalen Tumorstadiums beim Kolonkarzinom sollten Rekonstruktionen entlang der Längsachse des Tumors erstellt werden, da dies die diagnostische Genauigkeit erheblich verbessert. Ebenso führt eine geringe Schichtdicke < 5 mm zu einer signifikant besseren Beurteilung der Lokalausdehnung des Tumors als bei Schichtdicken > 5 mm [24].

## TNM-Stadieneinteilung

### Lokales Tumorstadium

**Abb. 1:**
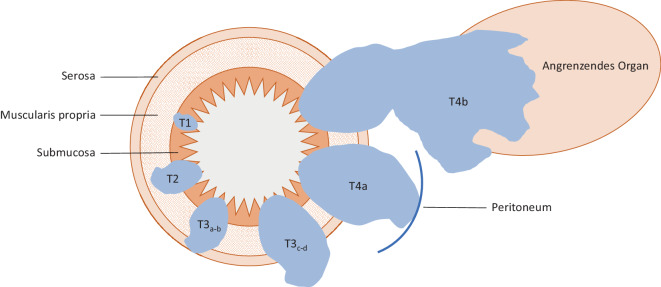
Radiologische TNM-Stadieneinteilung (mod. nach Chang et al. [9]).*T1* Karzinom in Submukosa, *T2* Karzinom bis in die Muscularis propria reichend, *T3*_*a–b*_ Karzinom bis an die Serosa bzw. bis < 5 mm über die Serosa hinausragend, *T3*_*c–d*_ > 5 mm über die Serosa hinausragend, *T4* Karzinom mit Infiltration des umgebenen Gewebes/Organs (*T4*_*a*_ Infiltration Peritoneum, *T4*_*b*_ Infiltration eines Organs). (Nach [28])

**Abb. 2:**
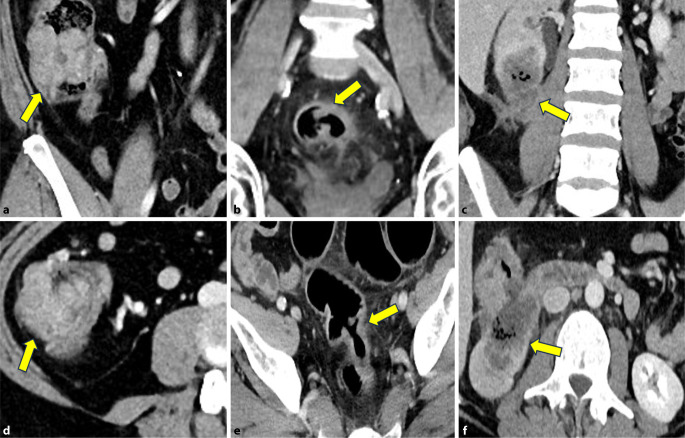
**a–c** Axial. **d–e** Koronal. **a,** **d** 56-jähriger Patient mit einem Karzinom im Zökum. Weder axial (**a**) noch koronal (**d**) zeigt sich eine Wandüberschreitung (T2 N0 M0, *gelbe Pfeile*). **b,** **e** 78-jähriger Patient mit einem Karzinom im Sigma. Vor allem auf der axialen Serie (**b**) zeigt sich eine deutliche Imbibierung des angrenzenden Fettgewebes (*gelbe Pfeile*) im Sinne einer Infiltration (T3ab N0 M0). **c,** **f** 63-jährige Patientin mit einem ausgedehnten Karzinom am Colon ascendens/Kolonflexur mit Infiltration der rechten Niere (T4b N0 M1, *gelbe Pfeile*)

Kolonkarzinome können sich entweder als polypoide Raumforderungen oder als (semi-)zirkumferente, fokale Verdickungen der Wand darstellen. Abb. 1 zeigt die lokale Stadieneinteilung der Kolonkarzinome. Es muss allerdings beachtet werden, dass T1- bis T2-Stadien mittels CT schwer zu unterscheiden sind und daher auch zusammengefasst werden. T1/2-Tumoren überschreiten die Muscularis propria (Abb. 2a, d) nicht. T3-Tumoren können in T3ab und T3cd unterteilt werden. T3ab-Tumoren infiltrieren die Serosa und überschreiten sie bis max. 5 mm (Abb. 2b, e). T3cd-Tumoren überschreiten die Serosa um mehr als 5 mm. T4a-Tumoren infiltrieren das Peritoneum und T4b-Tumoren infiltrieren angrenzende Organe (Abb. 2c, f). In Tab. 1 sind die genauen Definitionen der einzelnen Unterkategorien angegeben.

**Tab. 1:** TNM-Stadieneinteilung des Kolonkarzinoms. (Adaptiert nach AJCC TNM Staging und Chang et al. [9, 28])

TNM	Stadium	Interpretation
*T*	TX	Unzureichende Informationen für adäquate Beurteilung
	T0	Kein Anhaltspunkt für Karzinom
	Tis	Karzinom in situ
	T1	Karzinom in Submukosa
	T2	Karzinom in Muscularis propria
	T3	Karzinom in Serosa
	T3_a–b_	Karzinom überschreitet Serosa < 5 mm
	T3_c–d_	Karzinom überschreitet Serosa > 5 mm
	T4a	Karzinom infiltriert das Peritoneum
	T4b	Karzinom infiltriert angrenzende Organe
*N*	NX	Unzureichende Informationen für adäquate Beurteilung
	N0	Keine Infiltration lokoregionärer Lymphknoten
	N1	1–3 suspekte Lymphknoten
	N2	4–6 suspekte Lymphknoten
*M*	MX	Unzureichende Informationen für adäquate Beurteilung
	M0	Keine Metastasen
	M1	Fernmetastasen inkl. Peritoneum

*T* Tumor, *N* Node, *M* Metastase

Bei der Unterscheidung zwischen Tumoren im Stadium T1–2 („low risk“) und T3–4 („high risk“) besteht die höchste Sensitivität in der CT. In allen Studien wurde eine akzeptable Detektionsrate mit Sensitivitäten von bis zu 90 % von T3/4-Tumoren mittels CT festgestellt. Bei gleichzeitig niedriger Spezifität von 69 % von T3/4-Tumoren kommt es zu einer nicht außer Acht zu lassenden Über- oder Unterschätzung der einzelnen Tumorstadien.

Wichtig ist auch die genaue Lokalisation des Tumors, einerseits in welchem Abschnitt des Kolons er sich befindet und andererseits, ob er sich in einem Bereich mit viel umliegenden Fettgewebe befindet oder eventuell in einem Bereich, wo das Kolon direkt ans Peritoneum angrenzt. Entsprechend kann ein gleich großer, wandüberschreitender Tumor entweder ein T3- oder T4-Stadium (Infiltration des Peritoneums) aufweisen. Das Vorhandensein einer extramuralen vaskulären Infiltration (EMVI) ist für die Stadieneinteilung nach TNM zwar nicht relevant, es konnte allerdings gezeigt werden, dass eine EMVI die Überlebenswahrscheinlichkeit negativ beeinflusst, da es vermehrt zu Metastasierungen kommt. Eine EMVI sollte deshalb unbedingt auch im Befund vermerkt werden [14, 18, 19, 24, 26].

Ist es in der Koloskopie aufgrund eines stenosierenden Tumors nicht möglich, die proximal des Tumors gelegenen Dickdarmabschnitte zu untersuchen, sollte idealerweise eine CT-Kolonographie zur Identifizierung möglicher Zweitkarzinome durchgeführt werden [29]. Auch bei einer Standard-CT-Untersuchung sollte auf Zweitkarzinome geachtet werden, da diese nicht mittels Koloskopie ausgeschlossen werden konnten.

Um die Limitationen der radiologischen Stadieneinteilung zu verringern, sollten strukturierte Befunde bevorzugt werden. Es hat sich gezeigt, dass die strukturierte Befundung anhand eines Befundschemas signifikant mehr relevante Schlüsselwörter enthält als frei diktierte Befunde [22]. Insbesondere wird mehr auf das quantitative Ausmaß der Darmwandüberschreitung des Tumors in Millimetern, den Lymphknotenstatus sowie die allgemeine Klassifizierung nach TNM eingegangen [22]. Wenig überraschend steigt auch die Genauigkeit der Stadieneinteilung von Kolonkarzinomen mit der Erfahrung der RadiologInnen an. Für die Lymphknotenbeurteilung konnte jedoch keine signifikante Lernkurve ermittelt werden [14, 31].

### Beurteilung der Lymphknoten

Neben der lokalen Stadieneinteilung von Kolonkarzinomen ist auch die Beurteilung von potenziell malignen Lymphknoten eine weitere Herausforderung. Die Genauigkeit der CT ist mit einer Sensitivität und Spezifität von nur 71 und 67 % unzureichend [14, 15, 18]. Unterschiedliche Lymphknotencharakteristika wurden bisher untersucht: Messen des Kurz- oder Langachsendurchmessers, Berandung des Lymphknotens, Gruppierung von Lymphknoten (> 3 Lymphknoten), Heterogenität und Kontrastmittelaufnahme. Eine Aussagekraft hinsichtlich Malignität wird bei Stadium T3 jedoch nur der Heterogenität sowie einer irregulären Berandung zugesprochen. Weitere stadiumunabhängige Malignitätskriterien sind ein vergrößerter Kurzachsendurchmesser > 9 mm, zahlenvermehrte Lymphknoten sowie unabhängig von der Größe eine Gruppierung im Bereich der peritumoralen und mesenteriellen Gefäße [15]. Je nach Tumorlokalisation befinden sich lokoregionäre, maligne Lymphknoten entweder entlang der A. ileocolica, A. colica media oder der A. colica dextra, wobei sich die meisten Lymphknotenmetastasen perikolisch, also in direkter Nachbarschaft zum Tumor befinden. Weiter entfernte Lymphknotenmetastasen können sich bis zu den linksseitigen, supraklavikulären Lymphknoten (*Virchow-Lymphknoten*) ausbreiten.

### Fernmetastasierung und Peritonealkarzinose

Das Vorhandensein von Metastasen ist die Haupttodesursache bei kolorektalen Karzinomen. Die häufigsten Metastasen treten in der Leber, der Lunge und dem Peritoneum auf. Bei Diagnose haben bereits 20 % der PatientInnen Metastasen, meistens in der Leber. Dies wird als synchrone Metastasierung bezeichnet. Innerhalb der ersten 5 Jahre nach Erstdiagnose entwickeln bis zu 60 % der PatientInnen Metastasen (metachrone Metastasierung). Sollten Lebermetastasen vorhanden sein, muss zusätzlich zur CT-Untersuchung auch eine MRT der Leber mit hepatozytenspezifischem Kontrastmittel zur genauen Planung des chirurgischen Eingriffs durchgeführt werden [1, 23].

### Stadieneinteilung mittels Magnetresonanztomographie

Zur Verbesserung der Sensitivität der initialen Stadieneinteilung werden immer wieder auch andere Modalitäten diskutiert, allen voran die Magnetresonanztomographie (MRT). Die MRT wird bei der Stadieneinteilung der Kolonkarzinome, anders als beim Rektumkarzinom, nicht in der Routine eingesetzt.

Vorteile der MRT gegenüber der CT sind die bessere Weichteildifferenzierung, das Fehlen ionisierender Strahlung und die Abklärung von Fernmetastasen, insbesondere die höhere Genauigkeit bei der Abklärung kleiner Lebermetastasen < 2 cm mit einer Sensitivität von 95 % zu 60 % (Positronen-Emissions-Tomographie [PET]/CT; [8, 17]). Einschränkend bei einer Kolonuntersuchung ist jedoch die längere Untersuchungsdauer, sowohl für den Patientenkomfort, aber auch in Bezug auf Artefakte durch die Peristaltik des Kolons. Eine Verkürzung der Untersuchungsprotokolle und Eingrenzung des Untersuchungsgebiets wären Ansätze zur Problemlösung [6, 8, 11].

Trotz der besseren Weichteildifferenzierung ist die MRT allerdings bei der Beurteilung der lokalen Tumorausdehnung der CT nicht wesentlich überlegen, auch hier kommt es bei einer Sensitivität von 60–80 % zu einer Unter- und Überschätzung des Tumorstadiums [17, 25].

Dem gegenüber steht, dass die MRT in der Unterscheidung zwischen Low-risk- und High-risk-Tumorstadium, der Beurteilung von Lymphknotenmetastasen und dem Vorhandensein einer extramuralen vaskulären Infiltration (EMVI) der CT überlegen ist [6, 17].

### Stadieneinteilung mittels Hybridbildgebung

Die PET/CT oder PET/MRT ist ebenso wie die MRT nicht in den Leitlinien verankert. Die F18- Fluorodesoxyglukose(FDG)-PET/CT oder -PET/MRT kann zur initialen Stadieneinteilung bei Kontraindikationen für eine MRT oder CT, wie z. B. Kontrastmittelallergien zum Einsatz kommen [18]. Hierbei kann es allerdings zu falsch-negativen Befunden kommen. Diese resultieren aus Adenokarzinomen mit niedrigem Glukosemetabolismus oder großen Anteilen an Nekrosen. Auch bei Metastasen unter 10 mm kann es zu einer fehlenden Traceranreicherung kommen. Falsch-positive Befunde resultieren von reaktiven Lymphknoten oder inflammatorischen Prozessen wie Divertikulitis, Fisteln oder Abszessen [18].

Eine erhöhte Sensitivität der F18-FDG-PET/CT gegenüber der CT zeigt sich in der Diagnostik von Lebermetastasen, in der Beurteilung von T3/4-Tumoren und von EMVI [11, 18]. In Bezug auf die Lymphknotenbeurteilung zeigt sich keine signifikante Überlegenheit gegenüber der CT [25]. Insgesamt scheint die PET/CT eine zusätzliche Möglichkeit für eine Evaluierung des Therapieansprechens bzw. bei Verdacht auf ein Rezidiv zu sein. Sie zeigt jedoch keine signifikant bessere Beurteilung im Rahmen der initialen Stadieneinteilung im Vergleich zur CT [8, 11, 18].

## Rektumkarzinom

### Stadieneinteilung des Rektumkarzinoms

Anders als beim Kolonkarzinom, spielt die MRT bei der primären Stadieneinteilung des Rektumkarzinoms eine bedeutsame Rolle. Gründe hierfür sind einerseits die anatomische Lage und Größe des Rektums und andererseits die deutlich geringen Bewegungsartefakte im kleinen Becken. Die wichtigsten Punkte des radiologischen Befundes inkludieren die genaue Lokalisation und Ausdehnung des Tumors (Infiltration der mesorektalen Faszie, des Peritoneums oder umliegender Organe) sowie die Beurteilung der lokoregionären Lymphknotenstationen.

Die Bildgebung spielt hier eine zentrale Rolle in der individuellen Prognoseabschätzung und Therapieplanung, wobei eine Bandbreite an unterschiedlichen Therapieoptionen zur Verfügung steht. Zu den wichtigsten Verfahren zählen Operation, neoadjuvante Radiotherapie oder kombinierte Chemotherapie. Die genaue Beschreibung der Tumorausdehnung ist essenziell für die Planung von Operation oder Radiotherapie [4, 12].

Aufgrund der Wichtigkeit der MRT in der Therapieplanung wird, analog zur Stadieneinteilung beim Kolonkarzinom, die Verwendung von Befundvorlagen von Expertengremien empfohlen. Dadurch soll sichergestellt werden, dass alle relevanten Informationen im Befund vollständig erfasst sind und der Befundtext zugleich übersichtlicher und leichter lesbar ist [2, 7]. Die wichtigste Basis der primären Stadieneinteilung bildet die aktuelle, 8. Version der TNM-Klassifikation. Sie muss jedoch beim Rektumkarzinom um einige wichtige morphologische Merkmale erweitert werden, die nicht in der TNM-Klassifikation vorkommen, aber dennoch prognostisch relevant sind.

### MRT-Protokoll

Laut den aktuellen Empfehlungen der Europäischen Gesellschaft für Gastrointestinale und Abdominelle Bildgebung (ESGAR) sollte eine MRT des Rektums auf einem 1,5-T- oder 3‑T-Gerät unter der Verwendung einer Oberflächenspule durchgeführt werden. Bei den Fragen, ob die Applikation von Spasmolytika oder die endorektale Füllung mit Ultraschallgel notwendig sind, konnte kein Konsensus erreicht werden. Hierfür gibt es somit keine klare Empfehlung.

Das Protokoll sollte zumindest 2D-T2-gewichtete Sequenzen (Schichtdicke ≤ 3 mm) ohne Fettunterdrückung in 3 Ebenen (transversal, sagittal, koronal) sowie eine diffusionsgewichtete Sequenz beinhalten. Die Angulierung der T2-gewichteten Sequenzen sollte auf die Lage des Tumors abgestimmt werden. Bei tiefsitzenden Karzinomen wird zusätzlich die Durchführung einer koronalen T2-gewichteten Sequenz mit Angulierung parallel auf den Analkanal empfohlen, damit die Beziehung zum Sphinkterapparat oder dessen Beteiligung beurteilt werden kann. T1-gewichtete Sequenzen und kontrastmittelverstärkte Sequenzen werden als optional angesehen [2].

## Tumormorphologie, Lokalisation und Ausdehnung

### Morphologie

Rektumkarzinome entstehen typischerweise auf dem Boden eines vorher bestehenden Adenoms. Diese Entwicklung wird Adenom-Karzinom-Sequenz genannt und bildet die Grundlage der Vorsorgekoloskopie. Adenome können entweder polypoid wachsen und einen Stiel besitzen oder flach (sessil) entlang der Rektumwand wachsen. Polypoide Karzinome weisen in der Regel eine niedriggradige Malignität auf. Die Stelle, an der ein Karzinom der Rektumwand entspringt, ist für das lokale Tumorstadium und die Beurteilung einer möglichen Rektumwandüberschreitung relevant. Auch die Unterscheidung zwischen soliden und muzinösen Karzinomen ist wichtig, da muzinöse Karzinome eine schlechtere Prognose zeigen [5, 32]. Muzinöse Tumoren sind auf T2-gewichteten Bildern gut sichtbar, während die Identifizierung muzinöser Lymphknoten eine Herausforderung darstellt. T1-gewichtete Bilder sind hier für eine präzisere Beurteilung vorteilhaft.

### Lokalisation

**Abb. 3:**
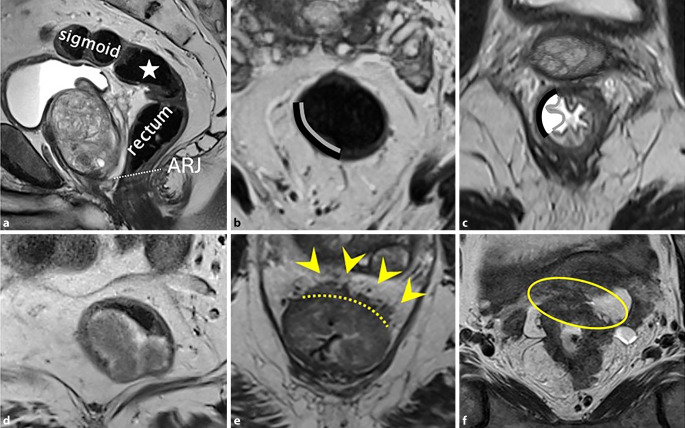
**a** Sagittale T2-gewichtete Aufnahme eines gesunden männlichen Rektums, die den Sigmoid-Rektum-Übergang (*Stern*) zeigt. Die *weiße*, *gestrichelte Linie* markiert die anorektale Übergangszone (*ARJ*). **b** Normale, zweischichtige Morphologie des Rektums in der MRT. **c** Im Vergleich dazu eine Rektumwand mit ödematös veränderten, dreischichtigen Aussehen (von innen nach außen: Mukosa, Submukosa, Muscularis propria) auf axialen T2-gewichteten MRT-Aufnahmen. **d** Ein 63-jähriger Patient mit einem polypoiden Tumor, der als Tumorstadium T1–2 beurteilt wird. Die Submukosa ist nicht eindeutig sichtbar, wodurch eine genaue Differenzierung zwischen T1 und T2 unmöglich wird. **e** Axiale T2-gewichtete Bilder eines Low-risk-T3ab-Tumors, der sich von etwa 12 bis 2 Uhr (*gelbe Pfeilspitzen*) über die Muscularis propria (*gestrichelte Linie*) hinaus erstreckt, mit einer extramuralen Invasionstiefe von unter 5 mm. **f** Eine 78-jährige Patientin mit einem cT4b-Rektumkarzinom, basierend auf der Invasion des Uterus (*gelber Kreis*)

Aufgrund der unterschiedlichen Behandlungsstrategien ist eine klare Differenzierung zwischen Kolonkarzinom, Rektumkarzinom und Analkarzinom essenziell, aber nicht immer einfach. Verschiedenste Definitionen und anatomische Orientierungspunkte wurden bereits in der Literatur beschrieben, wobei sich der Begriff des „sigmoid take-off“ in den letzten Jahren als Grenze zwischen Colon sigmoideum und Rektum durchgesetzt hat [5, 10]. Dieser Punkt lässt sich am einfachsten in der sagittalen, T2-gewichteten Sequenz abgrenzen. Sigma und Rektum lassen sich anhand ihres Verlaufs unterscheiden, wobei das Sigma horizontal weg vom Sakrum verläuft. Jeder Tumor, der sich oberhalb dieses Punkts befindet, wird als Sigmakarzinom bezeichnet, Tumoren darunter als Rektumkarzinom (Abb. 3a).

Die distale Begrenzung des Rektums hin zum Analkanal ist ebenfalls ein wichtiger Bestandteil der Befundung. Die makroskopisch sichtbare Grenze zwischen Rektum und Analkanal, die Linea dentata, ist radiologisch nicht abgrenzbar. Eine weitere, ca. 2 cm oral der Linea dentata gelegene Begrenzung stellt der anorektale Übergang dar (Abb. 3a). Hierzu kann in der sagittalen T2-gewichteten Sequenz eine imaginäre Linie zwischen dem Unterrand des Os coccygis und Os pubis eingezeichnet werden. Für die Therapieplanung sind zwei Punkte essenziell: einerseits das Vorhandensein einer Infiltration des Rektumkarzinoms in den Analkanal (und welchen Teil davon) und andererseits der Abstand zwischen Rektumkarzinom und Beginn des Analkanals. Der Abstand zum Analkanal bestimmt das operative Vorgehen und entscheidet, ob eine tiefe vordere Resektion und Anastomose durchgeführt werden kann.

### Ausdehnung

Die drei wichtigsten Schichten der Rektumwand sind die innen befindliche Mukosa, Submukosa und die umgebende Muscularis propria [3]. Bei der routinemäßigen MRT des Rektums erscheint die Wand normalerweise als zweischichtige Struktur, wobei die Muscularis propria ein hypointenses Signal zeigt (Abb. 3b). Die Mukosa und Submukosa sind in der Regel nicht unterscheidbar, es sei denn, es liegt ein submukosales Ödem vor, das ihre Differenzierung als getrennte Schichten ermöglicht (Abb. 3c). Das Tumorstadium („T“ innerhalb der TNM-Stadieneinteilung) wird anhand der Tiefenausdehnung der Tumorinfiltration innerhalb dieser 3 Schichten definiert. Das Tumorstadium ist ein wichtiger Faktor in der Entscheidung der besten, individuellen Behandlungsoption und zur Risikoabschätzung. Tumoren, die auf die Submukosa (T1) beschränkt sind oder sich maximal in die Muscularis propria ausdehnen, aber nicht darüber hinaus (T2), werden als Tumoren im Frühstadium bezeichnet. Diese können entweder endoskopisch abgetragen oder mittels totaler mesorektaler Exzision chirurgisch entfernt werden. Die begrenzte Sichtbarkeit der einzelnen Schichten der Darmwand ist ein entscheidender Faktor, warum in der MRT in der Regel nicht zwischen T1- und T2-Tumoren unterschieden werden kann, weshalb diese häufig gemeinsam als Stadium T1–2 befundet werden (Abb. 3d). Eine genaue Unterscheidung zwischen T1 und T2 ist durch eine ergänzende endorektale Ultraschalluntersuchung möglich. Wenn der Tumor die Muscularis propria durchbricht und das mesorektale Fettgewebe infiltriert, handelt es sich um einen T3-Tumor. Diese Tumoren können von Tumoren mit geringem Risiko und begrenzter extramuraler Invasion (T3a und T3b; Abb. 3e) bis hin zu Tumoren mit höherem Risiko und ausgedehnterer extramuraler Invasion (T3c und T3d) reichen. T3-Tumoren können auch die mesorektale Faszie (MRF) infiltrieren. T4-Tumoren zeigen eine Infiltration in angrenzende Strukturen oder Organe (Abb. 3f). Die Ausdehnung des Tumors entlang der Rektumwand wird wie auf einem Ziffernblatt einer Uhr bezeichnet (z. B. von 4–6 Uhr).

**Abb. 4:**
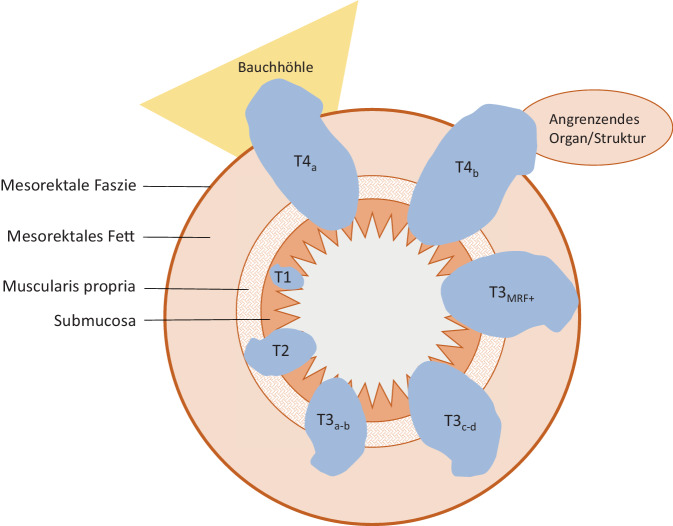
Radiologische TNM-Stadieneinteilung des Rektumkarzinoms. *T1* Karzinom infiltriert Submukosa, *T2* Karzinom infiltriert die Muscularis propria, *T3*_*a–b*_ Karzinom überschreit die Rektumwand und infiltriert das mesorektale Fettgewebe bis < 5 mm, *T3*_*c–d*_ Infiltration des mesorektalen Fettgewebes > 5 mm, *T4* Karzinom mit Infiltration des umgebenen Gewebes/Organs (*T4*_*a*_ Infiltration Peritoneum, *T4*_*b*_ Infiltration eines Organs)

Innerhalb der TNM-Stadieneinteilung gibt es keine Berücksichtigung einer Infiltration des Analkanals. Da das Ausmaß der Infiltration allerdings therapieentscheidend sein kann, wurde im Rahmen eines Expertenkomitees festgelegt, dass die Infiltration des M. sphincter ani internus zwar im Befund erwähnt werden soll, aber mit keiner Änderungen des T‑Stadiums einhergeht. Um Konsistenz zwischen radiologischen und pathologischen Befunden zu gewährleisten, wurde entschieden, dass eine Invasion des äußeren Analsphinkters, des M. puborectalis oder M. levator ani (Skelettmuskeln) als T4b klassifiziert werden sollte, da PathologInnen eine Invasion in Skelettmuskeln als pT4b betrachten [16]. Abb. 4 zeigt schematisch die stadienhafte lokale Ausdehnung des Rektumkarzinoms. Tab. 2 beinhaltet die genaue Auflistung der TNM-Kriterien des Rektumkarzinoms.

**Tab. 2:** TNM-Stadieneinteilung des Rektumkarzinoms

TNM	Stadium	Interpretation
*T*	TX	Unzureichende Informationen für adäquate Beurteilung
	T0	Kein Anhaltspunkt für Karzinom
	Tis	Karzinom in situ
	T1	Karzinom in Submukosa
	T2	Karzinom in Muscularis propria
	T3	Karzinom überschreitet die Rektumwand
	T3_a–b_	Karzinom infiltriert mesorektales Fettgewebe < 5 mm
	T3_c–d_	Karzinom infiltriert mesorektales Fettgewebe > 5 mm
	T4a	Karzinom infiltriert das Peritoneum
	T4b	Karzinom infiltriert angrenzende Organe/Strukturen
*N*	NX	Unzureichende Informationen für adäquate Beurteilung
	N0	Keine suspekten lokoregionären Lymphknoten
	N1a	1 suspekter Lymphknoten
	N1b	2–3 suspekte Lymphknoten
	N1c	Tumordeposit
	N2a	4–6 suspekte Lymphknoten
	N2b	≥ 7 suspekte Lymphknoten
*M*	MX	Unzureichende Informationen für adäquate Beurteilung
	M0	Keine Metastasen
	M1a	Metastasen in einem Organ (mehrere Metastasen in paarigen Organen, z. B. Lungen zählen ebenfalls als M1a)
	M1b	Metastasen in ≥ 2 Organen
	M1c	Infiltration des Peritoneums

*T* Tumor, *N* Node, *M* Metastase

### Mesorektale Faszie und Peritoneum

**Abb. 5:**
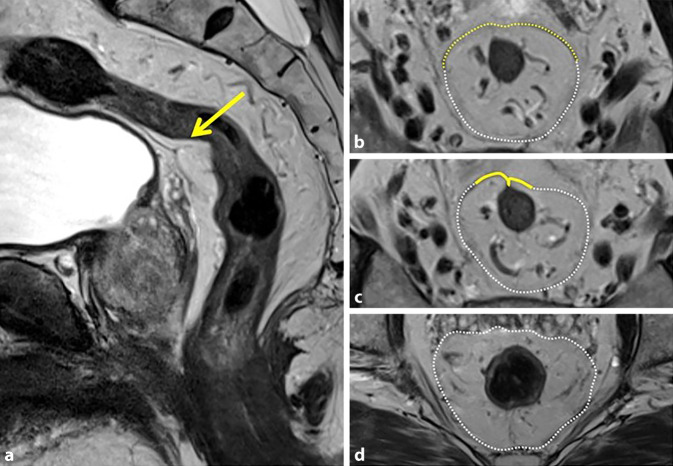
**a** Sagittale T2-gewichtete MRT-Bilder, welche die vordere peritoneale Umschlagsfalte bei einem Mann (ohne Rektumkarzinom) auf Höhe der Samenbläschen (*gelber Pfeil*) zeigen. **b** Auf der Ebene oberhalb und an der peritonealen Umschlagsfalte (**c**, *gelbe Linie*) steigt die mesorektale Faszie (MRF, *gestrichelte weiße Linie*) dorsolateral auf und bedeckt nur die lateralen und dorsalen Teile des Mesorektums, während das vordere Mesorektum vom Peritoneum (*gestrichelte gelbe Linie*) bedeckt ist. **d** Die MRF (*gestrichelte weiße Linie*) umgibt das gesamte Mesorektum

Als Mesorektum wird das umgebende Fettgewebe des Rektums bezeichnet. Hier finden sich lokale Lymphknoten, Lymphbahnen und Gefäße. Sobald das Rektumkarzinom die Muscularis propria durchbricht und das Mesorektum infiltriert, handelt es sich um einen T3-Tumor. Die mesorektale Faszie (MRF) ist eine zarte, fibröse Struktur, die das Mesorektum umgibt und auf der T2-gewichteten Sequenz als hypointense Linie abgrenzbar ist (Abb. 5d). Die Beurteilung der Infiltration der mesorektalen Faszie ist ein essenzieller Bestandteil des radiologischen Befundes. Eine Infiltration der MRF erhöht das Risiko eines Lokalrezidivs und erschwert eine komplette Entfernung des Tumors im Gesunden. Die aktuellen Richtlinien enthalten keine spezifischen Empfehlungen zur Stratifizierung von Patienten für eine neoadjuvante Therapie, basierend auf der MRF-Beteiligung. Das Gremium einigte sich darauf, dass die MRF als beteiligt gilt, wenn der Abstand ≤ 1 mm zum Primärtumor, zu unregelmäßig vergrößerten Lymphknoten, Tumordeposits oder EMVI beträgt. Glatt-vergrößerte Lymphknoten mit intakter Kapsel, welche die MRF berühren, sollten jedoch nicht als beteiligt angesehen werden [14]. Analog zur Ausdehnung des Karzinoms entlang der Rektumwand wird auch die Infiltration der MRF wie auf einem Ziffernblatt einer Uhr angegeben.

Die Abschnitte des mittleren und oberen Rektums sind ventral bis ventrolateral von Peritoneum überzogen, während die dorsalen Abschnitte und das untere Rektum extraperitoneal liegen und von der mesorektalen Faszie berandet sind. Auf der T2-gewichteten Sequenz ist die peritoneale Umschlagsfalte als zarte, hypointense Struktur abgrenzbar (Abb. 5a; [13]). Die Unterscheidung zwischen peritonealen und extraperitonealen Abschnitten des Rektums ist für die Stadieneinteilung essenziell: Eine Infiltration der MRF ergibt ein Lokalstadium T3 MRF+, wohingegen die Infiltration des Peritoneums ein Lokalstadium T4a ergibt mit einem erhöhten Risiko einer intraabdominellen Aussaat (Abb. 5b, c).

### Extramurale Gefäßinvasion

Kleinste venöse Gefäße befinden sich ausgehend von der Rektumwand im angrenzenden Fettgewebe. Bei wandüberschreitenden Tumoren kann es zu einer direkten, kontinuierlichen Fortsetzung von Tumorgewebe in diese kleinen Venen kommen, dies wird als extramurale Gefäßinvasion (EMVI) bezeichnet [27]. In der MRT erkennt man eine Gefäßinvasion entweder daran, dass das Gefäß eindeutig mit Tumorgewebe gefüllt ist und somit eine vergleichbare Signalintensität wie der Tumor aufweist, oder an einer fokalen Dilatation der Vene aufgrund von Tumorgewebe. Das Vorhandensein einer EMVI erhöht das Risiko eines Lokalrezidivs und von Metastasen.

### Lymphknotenstationen und Fernmetastasen

Das Vorhandensein und die Lokalisation von Lymphknotenmetastasen verändern Therapieoptionen und Risikoabschätzung. Obwohl Lymphknoten in der MRT nicht mit letzter Sicherheit als eindeutig maligne eingestuft werden können, gibt es einige Merkmale, die für das Vorhandensein einer Lymphknotenmetastase sprechen. Lymphknoten im Mesorektum werden bei einem Durchmesser ≥ 9 mm oder einer muzinösen Signalgebung immer als maligne eingestuft. Kleinere Lymphknoten benötigen zusätzlich eine suspekte Morphologie (rundlich, irregulär oder inhomogen), um als maligne charakterisiert werden zu können. Bei einem Lymphknoten zwischen 5 und 9 mm müssen zwei zusätzliche suspekte Merkmale vorhanden sein und bei einem Lymphknoten < 5 mm drei zusätzliche Merkmale. Bei den lateralen Lymphknoten (obturatorisch, entlang der A./V. iliaca externa und interna) wird lediglich anhand ihrer Größe entschieden, ob sie als suspekt zu werten sind. Eine retrospektive, multizentrische Analyse von 741 Patienten zeigte, dass obturatorische Lymphknoten und Lymphknoten entlang der A./V. iliaca interna bei einem Querdurchmesser ≥ 7 mm als Lymphknotenmetastase gewertet werden sollen, da diese Größe mit einem erhöhten Risiko für laterale Lymphknotenrezidive verbunden ist [20]. Obwohl eine weitere Validierung erforderlich ist, kann dieser Grenzwert vorerst verwendet werden.

Wichtig ist auch die Unterscheidung zwischen regionären und nichtregionären Lymphknoten. Zu den regionären Lymphknoten zählen Lymphknoten im Mesorektum, obturatorisch und entlang der V./A. iliaca interna. Ein Befall dieser Lymphnoten wird mittels N‑Stadium beurteilt. Zu den nichtregionären Lymphknoten zählen Lymphknoten entlang der A./V. iliaca communis und iliaca externa. Diese werden als Fernmetastasierung klassifiziert und mittels M‑Stadium abgebildet. Pathologische inguinale Lymphknoten werden prinzipiell als Metastasen klassifiziert, können jedoch bei distalen Tumoren unter Beteiligung des Analkanals aufgrund der lymphatischen Drainage zu den oberflächlichen inguinalen Lymphknoten als regionäre Metastasen (N-Stadium) betrachtet werden (Tab. 3).

**Tab. 3:** Zusammenfassung der unterschiedlichen Lymphknotenstationen in Relation zur Stadieneinteilung nach TNM

Regionäre LK (N-Stadium)	Nichtregionäre LK (M-Stadium)
Mesorektal	Entlang der A./V. iliaca communis
Obturatorisch	Entlang der A./V. iliaca externa
Entlang A./V. iliaca interna	Inguinal*

* Bei distalen Tumoren, die in den Analkanal eindringen oder dort hauptsächlich lokalisiert sind, können inguinale Lymphknoten aufgrund der lymphatischen Drainage aus dem Analkanal als regionale (N-Stadium) Lymphknoten betrachtet werden

Fernmetastasierung in ein Organ wird als M1a klassifiziert (zeigen sich Metastasen in paarig angelegten Organen, wird dies trotzdem als M1a klassifiziert, z. B. Metastasen in beiden Nieren). Fernmetastasen in zwei oder mehr Organen werden als M1b klassifiziert und jegliche peritoneale Metastasen als M1c, unabhängig davon, ob Organe zusätzlich betroffen sind.

### Strukturierter MRT-Befund Rektumkarzinom

Mehrere in der MRT beschriebenen Tumor- und Metastasenmerkmale beeinflussen die weiterführende Therapie und Behandlungsoptionen. Ein klar strukturierter MRT-Befund erleichtert die individuelle Therapieplanung und hilft Fehler zu vermieden.

Der strukturierte Befund sollte folgende Punkte beinhalten: genaue Lokalisation, Größe und Morphologie des Tumors. T‑Stadium, Beteiligung der mesorektalen Faszie und Beurteilung einer extramuralen Gefäßinvasion. Lokalisation der suspekten Lymphknoten inkl. N‑Stadium, Tumordeposits und Fernmetastasen.

## Fazit für die Praxis

- Bei Kolonkarzinomen ist eine Unterscheidung von T1/2-Tumoren und T3/4-Tumoren zuverlässig möglich, die genaue Stadieneinteilung mittels Computertomographie (CT) ist hingegen äußerst schwierig.
- Eine geringere Schichtdicke (< 5 mm) und multiplanare Rekonstruktionen sind essenziell.
- Bei stenosierenden Tumoren sollte immer nach Zweitkarzinomen gesucht werden.
- Suspekte Lymphknoten haben folgende Kriterien: vergrößerter Kurzachsendurchmesser (≥ 9 mm), Gruppierung, Heterogenität und Kontrastmittelaufnahme sowie eine irreguläre Berandung.
- Beim Rektumkarzinom ist die Magnetresonanztomographie (MRT) die wichtigste bildgebende Untersuchung zur radiologischen Stadieneinteilung.
- Neben den Kriterien der TNM-Klassifikation (Tumor, Node, Metastasen) sind noch weitere Merkmale zur individuellen Therapieplanung und Risikoabschätzung wesentlich: Infiltration des Peritoneums oder der mesorektalen Faszie und Abstand zum Analkanal bzw. Infiltration des Sphinkterapparats.
- Es wird zwischen regionären und nichtregionären Lymphknoten unterschieden, dementsprechend ändert sich auch die Stadieneinteilung.
